# Postpartum depression literacy in Chinese perinatal women: a cross-sectional study

**DOI:** 10.3389/fpsyt.2023.1117332

**Published:** 2023-06-14

**Authors:** Weijian Huang, Guiqin Li, Dongmei Wang, Hua Qu, Maozhou Tian, Yanmei Wang

**Affiliations:** ^1^Eastern Operating Room, Yantai Yuhuangding Hospital, Yantai, China; ^2^Obstetrics Department, Yantai Yuhuangding Hospital, Yantai, China; ^3^Obstetrics Department, Yantai Hospital of Traditional Chinese Medicine, Yantai, China; ^4^Department of Cardiac Surgery, Yantai Yuhuangding Hospital, Yantai, China; ^5^Hemodialysis Department, Yantai Yuhuangding Hospital, Yantai, China

**Keywords:** perinatal women, China, postpartum depression, mental health literacy, social support, self-efficacy

## Abstract

**Background:**

Postpartum depression literacy is a specific mental health literacy that can help perinatal women identify, manage, and prevent postpartum depression. However, the current status and associated factors of postpartum depression literacy among Chinese perinatal women are still unclear. This study investigated postpartum depression literacy and its associated factors among this group.

**Methods:**

A cross-sectional survey was conducted involving 386 cases of perinatal women using the convenience sampling method. Participants completed four questionnaires to evaluate their general characteristics, postpartum depression literacy, perceived social support, and general self-efficacy. The SPSS 24.0 software was used for descriptive statistical analysis, univariate analysis, and multivariate analysis.

**Results:**

The total PoDLiS score was (3.56 ± 0.32). The factors that composed the final multiple regression equation included planned pregnancy condition (*β* = −0.137, *p* = 0.003), education (*β* = 0.127, *p* < 0.001), history of depression (*β* = −0.271, *p* < 0.001), social support (*β* = 0.0012, *p* < 0.001), self-efficacy (*β* = 0.030, *p* < 0.001), and complications (*β* = −0.0191, *p* = 0.0019). They accounted for 32.8% of the total postpartum depression literacy variation (*R*^2^ = 0.328, *F* = 24.518, *p* < 0.001).

**Conclusion:**

The findings of this study improved our understanding of perinatal women’s postpartum depression literacy and its associated factors. Women with low postpartum depression literacy urgently need to be identified. Comprehensive nursing intervention measures should be taken from six dimensions of mental health literacy, social support, and self-efficacy to improve the postpartum depression literacy of perinatal women.

## Introduction

Postpartum depression (PPD) is a non-psychotic depressive episode that last more than 2 weeks after delivery (1). Women suffering from postpartum depression may depict appetite, sleep disturbance, fatigue, hostility, lower tolerance, difficulty concentrating, guilt, low self-esteem, and even suicidal thoughts (2). PPD can adversely affect the health of mothers (2), newborns (3), partners (4), and other family members (5).

PPD has a high incidence worldwide. A systematic review, which primarily included studies conducted in high-income countries, suggested that 19.2% of new mothers may suffer from depression in the first 12 weeks after giving birth (6). A meta-analysis of postpartum depression in low-and middle-income countries revealed that the prevalence of common non-psychotic postpartum psychiatric disorders was 19.8% (7). Other studies have attributed the differences in prevalence during the postpartum period (4.4–73.7%) to sampling methods, measurement tools, and socio-demographic differences throughout the study (8–10).

Despite the availability of treatments, few women actively seek professional help for symptoms of depression during the perinatal period. Fonseca et al. (11) found that only 13.6% of perinatal depressed women sought help for their emotional problems. The inability to recognize postpartum depressive symptoms, acquire information about the disease, and get self-care skills, as well as inadequate knowledge of treatment options, have been identified as the most important barriers to seeking professional help during the perinatal period (12–14).

Mental Health Literacy (MHL) is defined as “knowledge and beliefs about mental disorders that contribute to the identification, management or prevention of mental diseases” (15). This conceptual framework includes six elements of mental health literacy: (a) The ability to identify specific disorders or different types of psychological distress; (b) Knowledge and belief about risk factors and causes; (c) Knowledge and belief about self-help interventions; (d) Knowledge and belief about the availability of professional help; (e) Attitudes conducive to recognition and appropriate recourse; and (f) knowledge on how to seek mental health information (16). Previous studies have shown that increased understanding of mental health disorders, awareness of available help and treatment resources, and reduced stigma of mental illnesses often help promote mental health services and improve mental health outcomes (17).

The existing research on mental health literacy mostly focused on depression, anxiety, schizophrenia, and bipolar disorder, among others (18). Meanwhile, the research population of mental health literacy is mainly students, the elderly, health workers, and mental patients (19–21). Although such research may also have potential benefits for women during and after pregnancy, fewer studies have been aimed toward increasing maternal awareness of preventing or recognizing mental disorders and managing their symptoms.

The present study aims to evaluate the postpartum depression literacy level of Chinese perinatal women and to determine the factors related to postpartum depression literacy. The results of this study will provide information for the intervention strategy of postpartum depression literacy improvement plan for Chinese perinatal women.

## Theoretical framework

Andersen’s Behavioral Model of Health Services Use was utilized as the theoretical framework to guide this study. Previous studies often adopted Andersen health services use model to find the factors related to health service use (22–24). Recently, this model has been expanded to predict variables related to mental health literacy (25) because people with higher mental health literacy are more likely to seek professional help timely and reasonably when they encounter mental illnesses (20).

Andersen’s model was constructed to describe the general health service use of residents (26). It includes three aspects: predisposing, enabling, and need factors. Predisposing factors usually refer to individual characteristics. Enabling factors are resources or characteristics conducive to access to health services. The need factors reflect people’s awareness of their physical or psychological discomfort and need for medical help. Based on related literatures (6, 11, 12, 14, 26), we included possible related predisposing factors (age, religious belief, marital status, residence, pregnancy intendedness, pregnancy count, delivery count, perinatal period), enabling factors (educational status, monthly household income, employment status, medical insurance, history of depression, social support, self-efficacy), and need factors (complications, self-rated health status) to find the predictors of postpartum depression literacy among Chinese perinatal women.

## Methods

### Research design and data collection

This cross-sectional study was conducted in four tertiary hospitals in four cities in China, namely Yantai, Yichang, Lanzhou, and Shenyang. The four cities are located in eastern, southern, western, and northern China, respectively.

A convenience sample of Chinese women who underwent routine pregnancy check-ups, postnatal check-ups, childbirth and pregnancy training were recruited from obstetric clinics, obstetric in-patients department and pregnant women’s schools of four tertiary hospitals to participate in this study between February 2021 and June 2021. Research assistants in the four hospitals received two online training sessions to master the investigation procedures and requirements for this study.

Perinatal women were eligible for inclusion in the study if they were (a) at least 20 years old, (b) currently pregnant or had given birth in the past 12 months, (c) can understand and respond to the scale, and (d) provided informed consent for inclusion in the study. Perinatal women were excluded from this study if they (a) utilized assisted reproduction, (b) were suffering from severe pregnancy complications, or (c) experienced stillbirths, neonatal deformities, or newborns suffering from serious diseases.

According to the formula, the sample size should be between 10 and 20 times the number of variables (27). Given that there were 17 variables in this study and a 10% non-response rate, the total sample size should be 374. Women that satisfied the inclusion and exclusion criteria were informed of the goals of this survey at the beginning of the study. Those who voluntarily participated in this research filled out the informed consent form. The study recruited 400 women, 392 women completed the questionnaires, and six were removed because of missing data, resulting in 386 questionnaires in the final data analysis. Therefore, the response rate was 96.5%.

### Measures

#### Dependent variable

Postpartum Depression Literacy was measured using the Postpartum Depression Literacy Scale (PoDLiS) developed by Mirsalimi et al. (28). The Cronbach’α for this scale was 0.78. The Chinese version of the scale (PoDLiS-C) was translated, and its psychometric properties were tested among Chinese perinatal women (29). PoDLiS-C contains six factors and 31 items, including the ability to recognize postpartum depression, knowledge of postpartum depression risk factors and causes, knowledge and beliefs of postpartum depression self-care activities, knowledge and beliefs about postpartum depression professional help available, attitudes of appropriate help-seeking, and knowledge of how to seek information related to postpartum depression. Each item was evaluated on a 5-point Likert scale (1 = not at all to 5 = always). The total score of PoDLiS-C ranges from 31 to 155, with a higher score representing a higher level of postpartum depression literacy. Since each subscale has a different number of items, the raw scores for every subscale are summed, then divided by the item count in that subscale. Similarly, the total score for the PoDLiS-C is summed, then divided by the whole questionnaire’s item count. Both procedures give a score of 1–5 using the following formula. Score = Summing raw scores (or each subscale score)/Number of items (or each subscale items). For the PoDLiS-C, the omega coefficient is 0.894, and the content validity index (CVI) is between 0.80 and 1. Confirmatory factor analysis (CFA) determined the satisfactory construct validity of the PoDLiS-C, with the six-factor model explaining 60.76% of the total variance and demonstrating good model fit (likelihood ratio *χ*^2^/df, 1.003; goodness-of-fit index, 0.916; adjusted goodness-of-fit index, 0.901; comparative fit index, 0.999; incremental fit index, 0.999; root mean square error of approximation, 0.003; and standardized root mean square error of approximation, 0.0478).

#### Independent variables

Three sets of independent variables were included in this survey: eight predisposing factors, seven enabling factors, and two need factors.

The predisposing factors were age, religious belief, marital status, residence, pregnancy times, parity, pregnancy intendedness and perinatal period. The continuous variables were divided into three buckets and entered the regression analysis as: age (1 = 21–30, 2 = 31–40, 3 = 41–50), pregnancy times (1 = 1 time, 2 = 2 times, 3 = ≥3 times), parity (1 = 0 time, 2 = 1 times, 3 = ≥2 times). Religious belief (0 = do not have a religious belief, 1 = have a religious belief), marital status (0 = do not have a spouse, 1 = have a spouse), residence (0 = urban, 1 = rural), pregnancy intendedness (0 = planned pregnancy, 1 = unplanned pregnancy), and perinatal period (0 = pregnancy period, 1 = postpartum period) were dichotomously coded.

The enabling factors were educational status, employment status, monthly average income, history of depression, medical insurance, social support, and self-efficacy. Educational status (1 = junior school or below, 2 = high school/specialized secondary school, 3 = specialty/bachelor, 4 = postgraduate or above) were categorized into four groups and entered the regression analysis. Monthly average income (1 = insufficient <3,000¥, 2 = moderate 3,000–5,000¥, 3 = adequate >5,000¥) were divided into three buckets and entered the regression analysis. Medical insurance (0 = have insurance, 1 = Not have insurance), employment status (0 = unemployed, 1 = employed) and history of depression (0 = none, 1 = yes) were dichotomized.

Social support and self-efficacy entered the regression analysis as continuous variables. Social support was evaluated via the Perceived Social Support Scale (PSSS) (30) (Cronbach’s alpha = 0.941). PSSS includes 12 items and 3 dimensions (friend’s support, family support, and other supports). Each item was assessed on a 7-point Likert scale (1 = “very strongly disagree” to 7 = “very strongly agree”). The total score is between 12 and 84, and individuals with higher marks perceive more social support. The Chinese PSSS has good reliability as well as validity among the Chinese population (31, 32). The Cronbach’s *α* value of the PSSS was 0.93 in this study.

Self-efficacy was assessed via the General Self-efficacy Scale (GSES) (33). The original General Self-Efficacy Scale includes 10 items. Each item is rated on a 4-point Likert scale (1 = “not at all true” to 4 = “exactly true”). The total score ranges from 10 to 40, and a higher score indicates a higher level of general self-efficacy. The General Self-Efficacy Scale has been translated into Chinese and demonstrated good reliability and validity (34). Cronbach’α for this study was 0.873.

The need factors were self-reported health status and complications. Self-reported health status was assessed on a 5-point Likert scale (1 = very bad to 5 = very good) and was entered into the regression analysis as continuous variables. Complications (0 = with complications, 1 = without complications) were dichotomously coded.

## Statistical analysis

Descriptive analyses were conducted using means and standard deviations (SD), frequencies, and percentages of the measurement variables. Kolmogorov–Smirnov analysis was used to test the fit of the data to the normal distribution (*p* > 0.05). The socio-demographic characteristics and postpartum depression literacy of the participants were presented using univariate analysis. ANOVA, *t*-test, and correlational analysis were conducted to show the relationship between the predisposing, enabling, and need factors and postpartum depression literacy. The total score of postpartum depression literacy was used as the dependent variable, and statistically significant variables in univariate analysis and correlation analysis were used as independent variables for multiple linear regression analysis. Multicollinearity between independent variables was detected, and the significant factors related to postpartum depression literacy were determined via hierarchical linear regression. As per Andersen’s behavioral model, three regression models were tested. In the first model, postpartum depression literacy was regressed on predisposing factors. Enabling factors were added in the second model. The final model contained predisposing, enabling, and need factors. The data in this study were analyzed using SPSS 24.0. An alpha level of 5% (*p* < 0.05) was considered statistically significant.

## Results

### Socio-demographic characteristics

A total of 386 perinatal women participated in this study, The socio-demographic characteristics of the participants are presented in Table 1.

**Table 1:** Socio-demographic characteristics of the sample by postpartum depression literacy (*n* = 386).

Factor	Variables	Categories	Frequency (*n*)	Percentage (%)	MHL		*t/F post hoc* (Scheffe)
					*M*	SD	
Predisposing factor	Age (year)	21–30 (a)	137	35.5	3.49	0.45	4.142^*^
		31–40 (b)	235	60.9	3.59	0.42	
		41–46 (c)	14	3.6	3.32	0.56	a,c < b
	Religious belief	Yes	34	8.8	3.50	0.40	−0.546
		No	352	91.2	3.55	0.44	
	Marital status	With spouse	364	94.3	3.54	0.44	−0.647
		Without spouse	22	5.7	3.60	0.42	
	Residence	Urban	325	84.2	3.56	0.43	1.717
		Rural	61	15.8	3.46	0.45	
	Pregnancy times	1	183	47.4	3.53	0.50	0.172
		2	140	36.3	3.56	0.37	
		≥3	63	16.3	3.53	0.40	
	Parity	0	230	59.6	3.53	0.47	0.362
		1	138	35.8	3.57	0.38	
		≥2	18	4.7	3.57	0.36	
	Pregnancy intendedness	Planned pregnancy	306	79.3	3.59	0.41	4.165^***^
		Unplanned pregnancy	80	20.7	3.37	0.49	
	Perinatal period	Pregnancy period	332	86.0	3.54	0.43	
		Postpartum period	54	14.0	3.58	0.47	
Enabling factor	Educational status	Junior school or below (a)	21	5.4	3.27	0.50	11.808^***^
		High school/specialized secondary school (b)	62	16.1	3.36	0.42	
		Specialty/Bachelor (c)	269	69.7	3.58	0.40	
		Postgraduate or above (d)	34	8.8	3.80	0.54	d > c > b > a
	Employment status	Employed	289	74.9	3.57	0.43	2.178^*^
		Unemployed	97	25.1	3.46	0.46	
	Household monthly income (RMB)	Insufficient (<3,000)	39	10.1	3.47	0.54	1.274
		Moderate (3,000–5,000)	229	59.3	3.53	0.45	
		Adequate (>5,000)	118	30.6	3.59	0.38	
	Medical insurance	Has insurance	348	90.2	3.54	0.43	−0.279
		Not has insurance	38	9.8	3.56	0.48	
	History of depression	Yes	25	6.5	3.77	0.41	2.632^**^
		No	361	93.5	3.53	0.44	
	Social support (6)	Correlation with PoDLiS (*r* = 0.407^***^)			65.40	9.21	
	Self-efficacy (6)	Correlation with PoDLiS (*r* = 0.395^***^)			23.62	4.21	
Need factor	Complications	No	364	94.3	3.56	0.41	2.350^*^
		Yes	22	5.7	3.22	0.67	
	Self-reported health status	Very bad	4	1.1	3.52	0.29	1.177
		Bad	5	1.3	3.25	0.64	
		Fair	63	16.3	3.57	0.37	
		Good	187	48.4	3.57	0.43	
		Very good	127	32.9	3.50	0.47	

*p < 0.05, **p < 0.01, ***p < 0.001

Table 1 also displays the results of bivariate regression analysis, including *t*-test and ANOVA, to test the correlation between socio-demographic characteristics and postpartum depression literacy. Among the predisposing factors, age and planned pregnancy condition were significantly associated with postpartum depression literacy. Women aged 31–40 years revealed a higher level of postpartum depression literacy than those aged 21–30 and 41–50 years old (*F* = 4.142, *p* < 0.05). Women who planned their pregnancy had higher postpartum depression literacy than those with an unplanned pregnancy (*t* = 4.165, *p* < 0.001).

Among the enabling factors, the level of education, employment status, and history of depression were significantly associated with postpartum depression literacy. The higher the education level the women attained, the higher their postpartum depression literacy level (*F* = 11.808, *p* < 0.001). Employed women had higher levels of postpartum depression literacy than unemployed women (*F* = 2.187, *p* < 0.05). Women with a history of depression had a higher level of postpartum depression literacy than those without a history of depression (*F* = 2.632, *p* < 0.01).

Among the need factors, only complications had a significant association with postpartum depression literacy. Women with complications had lower postpartum depression literacy than those without complications (*t* = 2.350, *p* < 0.05).

### Postpartum depression literacy profiles

Among the variables explored in this study, age, pregnancy intendedness, education status, employment status, history of depression, and complications were significantly associated with postpartum depression literacy. Self-efficacy (*r* = 0.395, *p* < 0.001) and social support (*r* = 0.407, *p* < 0.001) were positively correlated with postpartum depression literacy.

Table 2 presents the total and subscale PoDLiS scores. The total PoDLiS score of perinatal women was 3.56 (0.32), indicating a moderate literacy level.

**Table 2:** Postpartum depression literacy scores of Chinese perinatal women (*n* = 386).

Dimensions	Number of items	Minimum	Maximum	Mean	SD	(%)	Sort descending
PoDLiS total score	31	1.65	4.81	3.54	0.44	70.8	
The knowledge and belief about postpartum depression professional help available	4	1.00	5.00	3.21	0.53	64.2	6
Knowledge of how to seek information related to postpartum depression	5	1.00	5.00	3.21	0.74	64.2	5
Ability to recognize postpartum depression	6	1.00	5.00	3.41	0.67	68.2	4
Attitude of appropriate help-seeking	6	1.00	5.00	3.51	0.61	70.2	3
Knowledge and belief of postpartum depression self-care activities	5	2.40	5.00	3.79	0.45	75.8	2
Knowledge of postpartum depression risk factors and causes	5	1.40	5.00	4.09	0.63	81.8	1

### Multivariate analysis of influencing factors of postpartum depression literacy

As detailed in Table 3, model 1 displays one of the predisposing factors, planned pregnancy or not (*β* = −0.227, *p* < 0.001) had significant effects on postpartum depression literacy, indicating the postpartum depression literacy of women carrying an unplanned pregnancy was lower than that of women with planned pregnancy, accounting for 4.1% of the variation (*F* = 9.260, *p* < 0.001).

**Table 3:** Hierarchical linear regression analysis on postpartum depression literacy (*n* = 386).

Factors variables	Model 1			Model 2			Model 3		
	*B*	SE	*p*-value	*B*	SE	*p*-value	*B*	SE	*p*-value
Predisposing									
Age (years) (1–3)	0.044	0.041	0.282	−0.020	0.035	0.574	−0.023	0.035	0.519
Planned pregnancy or not (ref = Planned pregnancy)	−0.227	0.054	0.000	−0.124	0.046	0.008	−0.137	0.046	0.003
Enabling									
Education (1–4)				0.127	0.029	0.000	0.127	0.029	0.000
Employment status (ref = employed)				−0.057	0.044	0.190	−0.051	0.044	0.242
History of depression (ref = yes)				−0.267	0.076	0.000	−0.271	0.076	0.000
Social support (cont.)				0.032	0.005	0.000	0.030	0.005	0.000
Self-efficacy (cont.)				0.013	0.002	0.000	0.012	0.002	0.000
Need									
Complications (ref = No)							−0.191	0.081	0.019
Coefficient	3.744	0.095	0.000	2.378	0.247	0.000	2.674	0.276	0.000
*F*	9.260		0.000	26.913		0.000	24.518		0.000
*R* ^2^	0.046			0.333			0.342		
Adjusted R^2^	0.041			0.320			0.328		

In model 2, in addition to the predisposing factor, planned pregnancy or not (*β* = −0.124, *p* = 0.008), there were three enabling factors, education level (*β* = 0.127, *p* < 0.001), social support (*β* = 0.013, *p* < 0.001), and self-efficacy (*β* = 0.032, *p* < 0.001) exhibited significant positive effects on postpartum depression literacy. One enabling factor，history of depression (*β* = −0.267, *p* < 0.001) had significant effects on postpartum depression literacy，indicating the literacy of perinatal women without a history of depression was lower than that of women with a history of depression. Model 2 explained 32.0% of the variation (*F* = 26.913, *p* < 0.001).

Among the three factors and eight variables of model 3, planned pregnancy or not (*β* = −0.137, *p* = 0.003), education level (*β* = 0.127, *p* < 0.001), history of depression (*β* = −0.271, *p* < 0.001), social support (*β* = 0.0012, *p* < 0.001), self-efficacy (*β* = 0.030, *p* < 0.001), and complications (*β* = −0.0191, *p* < 0.005) were confirmed to be significant factors in determining postpartum depression literacy levels. The final model explained 32.8% of the variance in postpartum depression literacy.

## Discussion

This study investigated the level of postpartum depression literacy and its influencing factors among a sample of Chinese perinatal women. Overall, the self-reported postpartum depression literacy level of the participants was moderate. The standardized, average score of postpartum depression literacy was (3.54 ± 0.44), and the total score was (109.86 ± 13.57), which was higher than that of Indian postpartum women (78.73 ± 11.82) (35) and lower than that of Iranian perinatal women (3.79 ± 0.39, 28). This discrepancy may be explained by differences in the ages, cultural backgrounds, and religious beliefs of the participants.

As illustrated in Table 2, “knowledge and beliefs about postpartum depression professional help available” received the lowest score at 64.2%. Gong et al. (36) found most of Chinese women with high-risk of perinatal depression said that they could cope with this depression without professional help. Jorm (16) suggested that individuals should be aware of the various types of mental health services available to obtain proper professional help, such as antidepressant medication and cognitive behavioral therapy. However, Goodman and Tyer-Viola (37) found that few women can compare the effects and benefits of different perinatal depression treatment methods. There is an urgent need for perinatal women to obtain professional help knowledge for postpartum depression. In this work, nurses in obstetric clinics can play a unique and important role.

The results of this study demonstrated that the score for “knowledge of information related to postpartum depression” was also very low, at 64.2% (Table 2). How individuals obtain information about mental disorders refers to which sources of information are used and whether the information provided by those sources is accurate. People commonly learn about mental disorders by reaching out to friends and family members who have experienced them in the past. According to Jorm (16), mass media is also an important source of information. Li et al. (38) found that PPD applications in China lack known effective intervention content. Accordingly, mental health professionals, including nurses, community health care workers, obstetricians and psychologists should be involved in the development of mobile applications for postpartum depression literacy based on evidence-based guidelines to ensure the accuracy and richness of the information.

Table 2 displayed that the score for “ability to recognize postpartum depression” was relatively low at 68.2%. Whitton found that “although 97% of women admit that they feel different from normal, only 25% think they may suffer from postpartum depression” (39), which reveals the difficulty for perinatal women to distinguish whether their emotional experience is normal stress or depression during the transition to being a parent (40). Due to the traditional custom of “doing the month” rooted in Chinese culture, a socially prescribed period of rest, women are confined to their homes and their activities are severely restricted. Meanwhile, the mother or mother-in-law are responsible for the health of puerperae and their newborns (41, 42). During this period, caregivers attach importance to maternal physical conditions and neglect their mental health, which may affect the identification of postpartum depression. However, awareness and recognition of postpartum depression symptoms have been considered the first step of the help-seeking process (43). Therefore, healthcare institutions should pay attention to the popularization of knowledge of postpartum depression symptoms and perform preliminary screening services for postpartum depression when necessary to promote the early diagnosis of postpartum depression.

The score for “attitudes of appropriate help-seeking” was 70.2%, indicating that perinatal women had a high level of stigma towards postpartum depression. Stigma is a process of labeling and stereotyping. For example, women with postpartum depression are considered violent, unpredictable, and dangerous (44). These negative prejudices will lead to high shame, low self-esteem, poor drug compliance, and other manifestations of people with mental symptoms. They will also hinder the people who are experiencing the prodrome of mental illness from seeking help and delaying treatment, thus reducing the overall recovery degree and quality of life and leading to disability or death in severe cases (45). WHO surveys illustrated that only 33% of the public seek professional help when experiencing psychological disorders (46). Therefore, medical staff should strive to eliminate the negative perception and attitude toward postpartum depression, dispel the stigma of postpartum depression, and encourage women with postpartum depression to seek professional help.

The results of hierarchical regression analysis guided by Andersen’s behavioral model revealed that one predisposing factor (pregnancy intendedness), four enabling factors (education, history of depression, social support, and self-efficacy), and one need factor (complications) were significantly associated with postpartum depression literacy among Chinese perinatal women.

In terms of predisposing factors, this study found that pregnancy intendedness was significantly associated with the postpartum depression literacy of perinatal women. This finding mirrored a previous study on knowledge and attitudes related to pre-pregnancy care in women with diabetes (47). Women carrying a planned pregnancy have usually made full preparations for their physical health, psychology, knowledge, and even the whole family, thus improving their postpartum depression literacy level (48). Meanwhile, some women who have unintended pregnancies have not fully prepared and often worry about their newborn’s health. Due to inadequate preparation, the birthing may disrupt the original pace of work and life, leading to various contradictions, and they often fail to account for the knowledge of postpartum depression (49). Therefore, healthcare providers should focus on postpartum depression literacy training for women with unplanned pregnancies to help them fully grasp the mental health literacy necessary for the arrival of a new life.

Among the enabling factors, a history of depression was identified as the most significant predictive factor, given the size of the variable coefficients in the regression model. This study revealed that women who suffered from psychiatric illnesses had higher postpartum depression literacy scores than their counterparts. The finding concurred with the results of previous studies (50, 51). Fonseca suggested that women with a psychiatric history can better identify symptoms of perinatal depression and are less likely to minimize the symptom due to their previous experiences with psychiatric disorders (11). Therefore, it is necessary for healthcare providers to invite perinatal women with a history of depression to share their experiences in coping with depression with perinatal women without a history of depression to achieve the goal of comprehensively improving postpartum depression literacy in their community.

Education was another enabling factor correlated with postpartum depression literacy. The results demonstrated that more educated women were more likely to have higher levels of postpartum depression literacy. This finding echoed the results of Reavley et al. (52), who reported that students at higher education levels were associated with correct recognition of depression. However, this is inconsistent with the finding of Piper et al. (20) among adults aged 60 and over, which may reflect the difference in age range between the sample of the current study (30+ years) and that of Piper (60+ years). Differences in education levels may only be related to the mental health of a younger sample. In other words, the social values and attitudes of younger groups may differ from those of older groups, and these values may persist over time despite social or cultural changes. It is suggested that medical personnel should introduce the topic of postpartum depression literacy in pregnancy and childbirth education and adopt various forms and easy-to-understand educational means to improve the postpartum depression literacy of perinatal women according to the differences in their education level.

The third enabling factor, social support, was positively correlated with postpartum depression literacy score, suggesting women with more social support were more likely to have higher postpartum depression literacy. The findings agreed with other studies that social support could promote the recognition of mental health problems, help-seeking attitudes, perceived barriers, and help-seeking intentions and reduce mental illness stigma (53, 54). It may even reduce the incidence of postpartum depression (55). Therefore, a mental health service system for pregnant women should be established, and a multidisciplinary team composed of maternal healthcare workers, mental health service providers, and social workers should be grouped to perform a collaborative care mode and provide mental health literacy training for pregnant women. At the same time, family members should learn about postpartum depression literacy to provide adequate support for maternal women, relieve their psychological stress, detect symptoms of postpartum depression, reduce the stigma of postpartum depression, and actively seek timely professional help.

Among the need factors, this study revealed that complications were significantly associated with postpartum depression literacy. Women with high EPDS scores had higher postpartum depression literacy. This finding did not support Kim’s result (25), which may be related to women getting more attention during pregnancy and childbirth in the context of Chinese culture. Therefore, when women or their family members recognize depressive symptoms, they often search for relevant knowledge through the internet, thus increasing postpartum depression literacy.

## Conclusion

The overall results of this study revealed average to high levels of postpartum depression literacy among the sample of Chinese perinatal women. The score rates of some dimensions were low, and the scores were not evenly distributed among dimensions. The demographic and maternal-related factors, such as pregnancy intendedness, educational background, history of depression, and complications, significantly influenced the level of postpartum depression literacy among Chinese perinatal women. This study identified two key influencing factors: social support and self-efficacy. Increased social support and self-efficacy correlated with improved postpartum depression literacy in perinatal women. It is imperative that the nursing profession addresses support, strategies, and solutions that may promote postpartum depression literacy in perinatal women.

## Limit

This study revealed important information about postpartum depression literacy among Chinese perinatal women. However, it has several limitations.

First, Due to the cross-sectional design and the convenience sampling strategy, the general applicability of the findings is limited.

Second, even though participants completed the questionnaire confidentially, there remains a possibility that some participants responded to items in a manner that they considered more socially acceptable.

Future studies should acknowledge these limitations and include other recruitment methods to obtain a bigger and more sociodemographically diverse sample. Further research should also examine the association between the person’s degree of closeness with PPD and knowledge and attitudes about it, as well as the role of public education campaigns on knowledge and attitudes about PPD, using a longitudinal design.

Therefore, postpartum depression literacy education should be implemented for pregnant women to improve maternal mental health literacy.

## Data availability statement

The raw data supporting the conclusions of this article will be made available by the authors, without undue reservation.

## Ethics statement

The studies involving human participants were reviewed and approved by Yan Tai Yuhuangding Hospital. The patients/participants provided their written informed consent to participate in this study.

## Author contributions

WH, HQ, YW, and MT designed the study and wrote the research protocol. WH, GL, DW, and MT did literature review, managed the field survey, quality control, statistical analysis, and prepared the manuscript draft. WH, YW, and MT contributed to the revisions in depth for the manuscript. WH and YW supervised the survey and checked data. All authors contributed to the article and approved the submitted version.

## Conflict of interest

The authors declare that the research was conducted in the absence of any commercial or financial relationships that could be construed as a potential conflict of interest.

## Publisher’s note

All claims expressed in this article are solely those of the authors and do not necessarily represent those of their affiliated organizations, or those of the publisher, the editors and the reviewers. Any product that may be evaluated in this article, or claim that may be made by its manufacturer, is not guaranteed or endorsed by the publisher.
